# Cyclical loading causes injury in and around the porcine proximal femoral physeal plate: proposed cause of the development of cam deformity in young athletes

**DOI:** 10.1186/s40634-015-0022-4

**Published:** 2015-03-08

**Authors:** Páll Sigurgeir Jónasson, Lars Ekström, Hans-Arne Hansson, Mikael Sansone, Jón Karlsson, Leif Swärd, Adad Baranto

**Affiliations:** Department of Orthopaedics, Institute of Clinical Sciences at Sahlgrenska Academy, University of Gothenburg, and Sahlgrenska University Hospital, Gothenburg, Sweden; Orthocenter/IFK-Kliniken, Gothenburg, Sweden; SportsMed, Gothenburg, Sweden; Institute of Biomedicine, Sahlgrenska Academy, University of Gothenburg, Gothenburg, Sweden

**Keywords:** Hip, Adolescent, Athlete, Physeal plate, Porcine, Biomechanics, Cam, Femoroacetabular impingement

## Abstract

**Background:**

The repetitive load to which the adolescent athlete’s body is exposed during training and competition affects bone growth. In previous studies, abnormalities of the spine and extremities of adolescent athletes have been described on radiographs and this also applies to the hip. The cam deformity of the hip is an extension of the physeal plate and develops during the adolescent athlete’s growth. Studies of the porcine spine have shown that the vertebral endplates, apophyseal rings and intervertebral discs are susceptible to both static and repetitive loads. The proximal physeal plate of the porcine femur is susceptible to static loads, but no studies have been performed on its susceptibility to repetitive loads. The purpose of this study was to investigate the susceptibility of the proximal porcine femur to repetitive loads.

**Methods:**

Descriptive laboratory study. Seven proximal femurs from four young (5 months) pigs were loaded repetitively (50,000 cycles) using a previously developed model. Three were loaded vertically, three antero-superiorly and one was used as a control. All femurs were examined macroscopically, histologically and with MRI after loading.

**Results:**

No macroscopic injuries were detected on any of the femurs after loading. Fluid redistribution was seen in all femurs on MRI compared with the unloaded control. Injuries were seen in all loaded femurs on microscopic examination of histological samples. Injuries, perpendicularly to the physeal plate and fractures adjacent to the plate, were seen in the vertically loaded specimens. In the antero-superiorly loaded specimen, the injury in the growth plate was parallel to the plate.

**Conclusion:**

Repeated loading of the young porcine hip leads to histological injuries in and adjacent to the physeal plate. These injuries are likely to cause growth disturbances in the proximal femur. We propose that such injuries may be induced in adolescent athletes and offer a plausible explanation for the development of the cam deformity.

## Background

For normal bone growth, physiological load is required (Malina 1969). Exceeding the normal physiological load may result in growth disturbances and unwanted morphological changes in the bone and joints (Caine et al. 2006; Strobino et al. 1952).

Abnormalities in the vertebral endplates, apophyseal rings and intervertebral discs have been demonstrated in the adolescent athlete participating in sports exposing the spine to high loads (Lundin et al. 2001; Sward et al. 1991). Abnormalities in the upper and lower extremities have also been reported in adolescent athletes (Caine et al. 2006). The etiology of the cam deformity of the femoral head-neck junction is multifactorial (Siebenrock and Schwab 2013). There is increasing evidence that the cam deformity can develop during growth periods in adolescent athletes (Siebenrock et al. 2011; Agricola et al. 2014).

**Figure 1:**
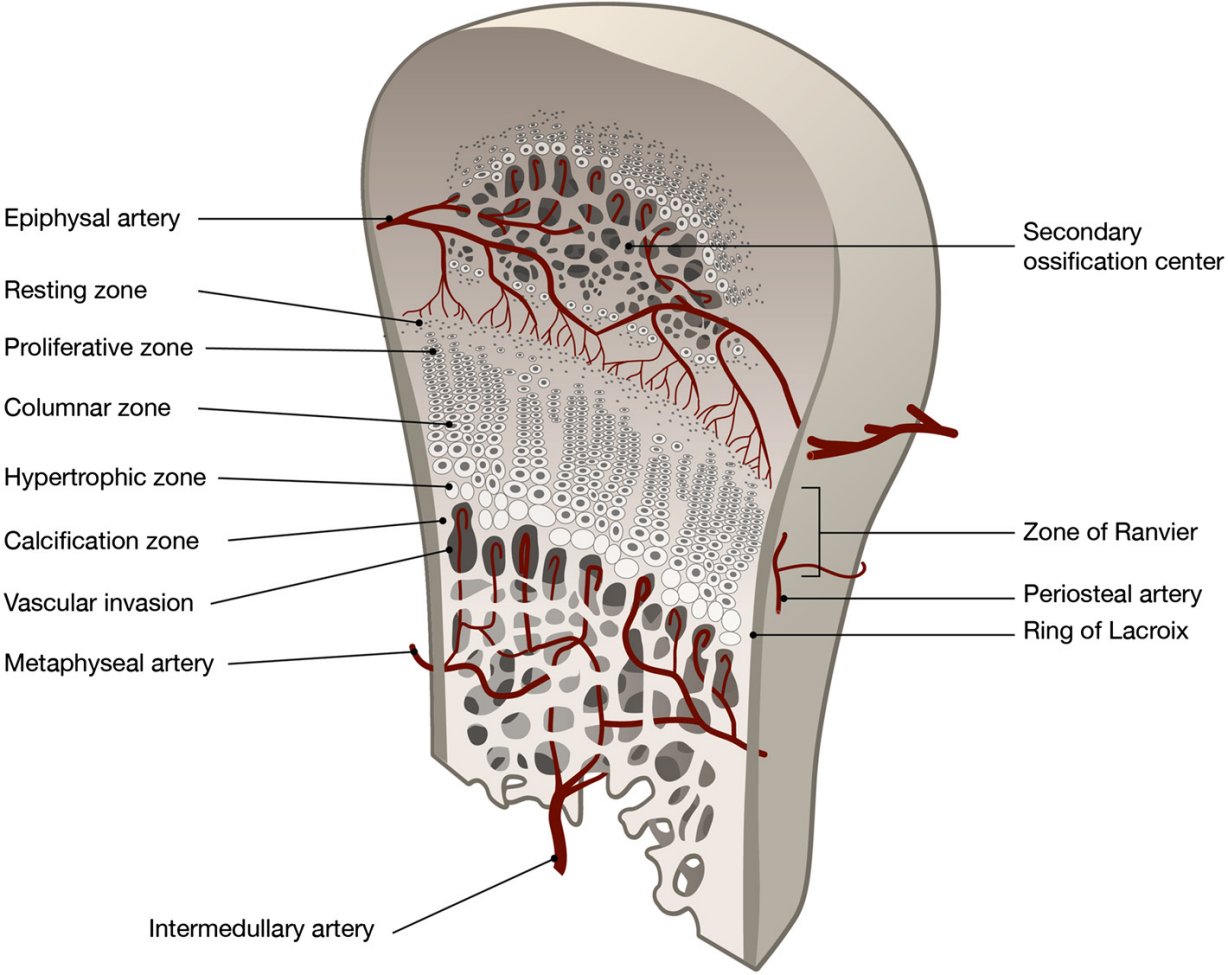
**Anatomy and blood supply of the physis.** The physis has been expanded to visualize the different cellular zones.

Repetitive loading during the performance of sports may lead to injuries to the physeal plate and growth interruption in young athletes. The microtrauma and occasional macrotrauma to which the adolescent athlete’s growing body is exposed affect the growing bone and joints. There are two ways this growth interruption can possibly occur; either directly, because of damage to the growth plate, or indirectly, because of interruptions to the growth plate blood supply. The growth plate blood supply is mainly derived from the epiphyseal side, through the epiphyseal artery, or the metaphyseal side, through the metaphyseal artery (Figure 1). Interruption of the epiphyseal blood supply causes narrowing of the growth plate and eventually growth cessation. Interruption of the metaphyseal blood supply causes widening of the physeal plate and may weaken it, but it does not cause growth cessation (Trueta and Amato 1960).

In a previous study, Jónasson et al. created an *in-vitro*, porcine model in which the effect of different loads and loading directions on the porcine proximal femur was investigated (Jonasson et al. 2014). The physeal plate was shown to be the weakest link in the proximal femur and especially susceptible to shearing forces. High loads to failure on the proximal femur resulted in physeolysis or fracture combined with physeolysis in all specimens.

The growth zone and, to a lesser extent, the endplate, is the weakest part of the young porcine lumbar spine when loaded in flexion, extension or axially (Baranto et al. 2005a; Baranto et al. 2005b). Biomechanical studies investigating the effect of cyclical loading and then axial compression to failure of the porcine spine have disclosed that the endplate and the growth zone are the weakest part of the cyclically loaded functional spinal units (Thoreson et al. 2010). There are no previous biomechanical studies investigating the effect of cyclical loading on the porcine proximal femur.

The purpose of the present study was to use the previously established model to investigate the effect of cyclical, sub-maximum loading on the porcine proximal femur. The hypothesis was that sub-maximum repetitive loading of the young porcine proximal femur would cause injuries in the physeal plate or in the epiphyseal or metaphyseal bone, adjacent to the physeal plate.

## Methods

### Experimental animals and procedures

Seven proximal femurs from four young (5 months) porcine hips were dissected and carefully cleaned of soft tissue, muscle and capsular attachments. The subjects were male domestic pigs, retrieved from the local abattoir, with a median weight of 90 kg (range 85–95). The diameter of the femoral heads was measured in the coronal and sagittal planes with a measurement error of less than 1 mm using a slide calliper (Kosashvili et al. 2008).

### Mechanical test procedures

One femur was used as a control. Six femurs were used for the mechanical testing procedures. The control was treated in exactly the same way as the other specimens bar the loading procedure.

**Figure 2:**
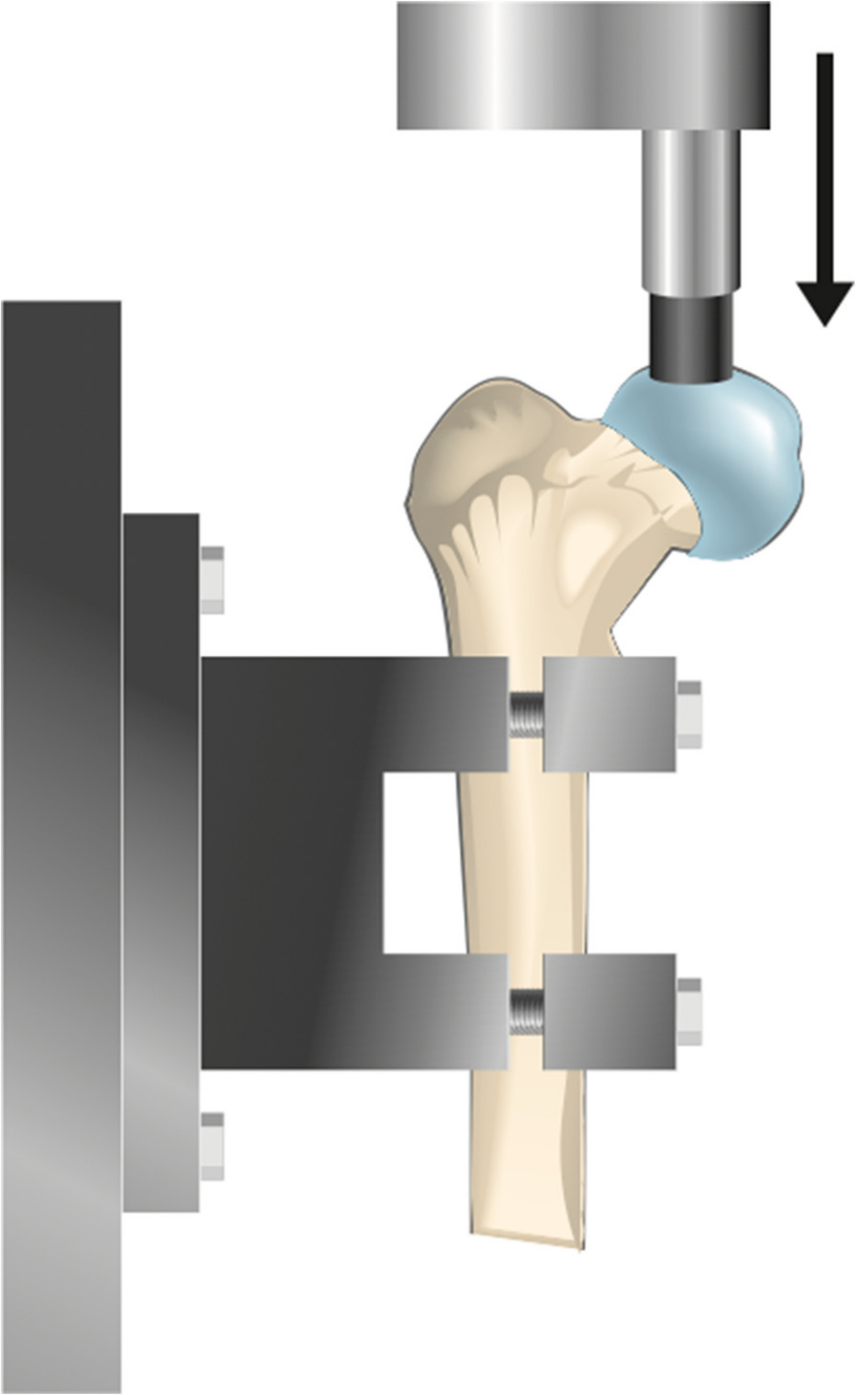
**Setup for vertical loading of the specimens.**

**Figure 3:**
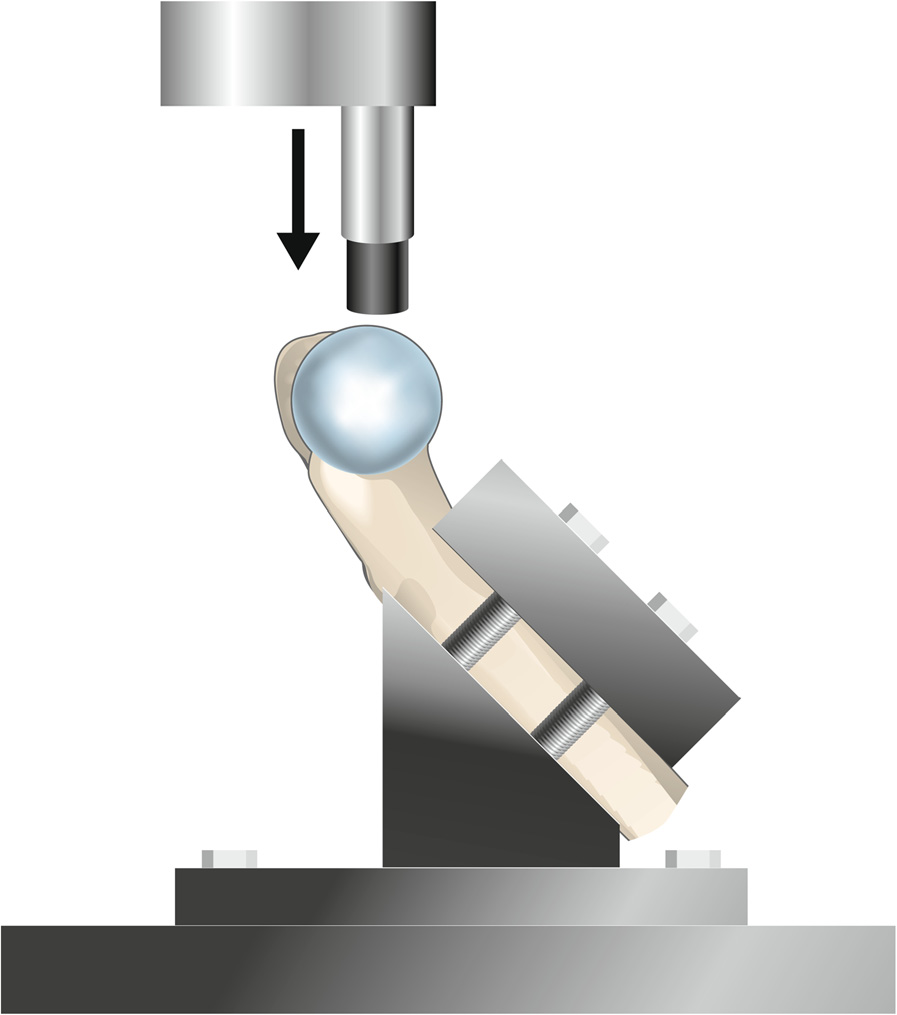
**Setup for antero-superior loading of the specimens, simulating 45° of flexion.**

The prepared proximal femurs were fixed with Plastic Padding™ (Loctite Technology) in a metal fixture and attached to the actuator of a servo hydraulic universal testing machine (MTS Test Star, Minneapolis, MN, USA). A custom-made aluminium rod with a small plastic cup (25 mm in diameter), contoured to the shape of the femoral head, was attached to the upper crosshead of the testing machine. The femurs were then loaded repetitively at 2 Hz for 50,000 cycles. Two Hz represents a good compromise between strain rate and exposure time in order to minimise the impact of dehydration. It is also closer to a normal running (1.5 Hz) cadence as opposed to a faster loading set-up. Fifty thousand cycles is equal to running four marathons (Thoreson et al. 2010). The number of cycles was used to correspond to a weekly cumulative load exposure during sport activities, e.g. long-distance runner and football players training between 11 and 16 hours a week. Three femurs (labelled 1–3) were loaded vertically with 2,500 N (Figure 2) and three femurs (labelled 4–6) were loaded with 2,000 N antero-superiorly (Figure 3), simulating 45° of hip flexion.

### Macroscopic examination

The physeal plate was clearly visible on the surface of the femoral head and was used as a guide for the placement of the plastic cup, proximally to the physeal plate. After loading, the sizes of the footprint left by the plastic cup on the femoral head and its distance from the physeal plate were measured. Any signs of epiphyseolysis or fracture, visual or palpable, were registered, if present.

### Magnetic resonance imaging

**Figure 4:**
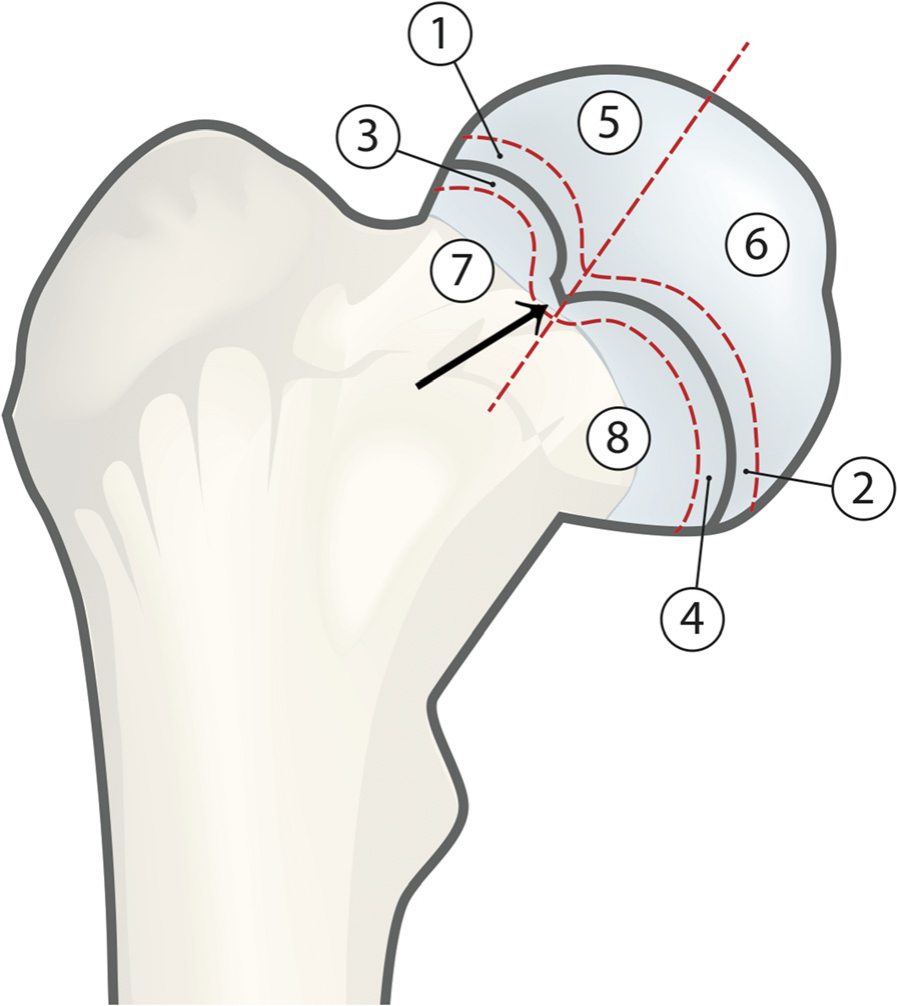
**The different zones compared on the MRI of the control to the MRI of the loaded specimens. 1** = Superior and adjacent to the physeal line, laterally to the epiphyseal tubercle (Black arrow). **2** = Superior and adjacent to the physeal line, medially to the epiphyseal tubercle. **3** = Inferior and adjacent to the physeal line, laterally to the epiphyseal tubercle. **4** = Inferior and adjacent to the physeal line, medially to the epiphyseal tubercle. **5** = Epiphysis, laterally to the epiphyseal tubercle. **6** = Epiphysis, medially to the epiphyseal tubercle. **7** = Metaphysis, laterally to the epiphyseal tubercle. **8** = Metaphysis, medially to the epiphyseal tubercle.

Directly after loading, Magnetic Resonance Imaging (MRI) was performed on all specimens, including the control femur, in a Philips Achieva 3.0™ (Philips Healthcare Inc.) using a wrist protocol, an extremity coil and SPAIR sequences. The MRI from each specimen was compared with the control and the difference in signal intensity was registered in eight different zones (Figure 4), medially and laterally to the epiphyseal tubercle in the epiphysis and the metaphysis and adjacent to the physeal plate, above and below.

### Microscopic and histological examinations

The specimens were then stored in a −20°C freezer until completely frozen. When frozen, they were sawn into four equally thick primary slices in the coronal plane using a band saw. The slices were numbered from one to four, with slice number one as the most anterior and slice 4 the most posterior. The slices were decalcified, dehydrated, fixed in paraffin and cut in 4 μm slices using a microtome. Four slices from each specimen were then stained with hematoxylin-eosin and alcian blue solution and the histological slices were examined microscopically for injuries by one of the co-authors, an experienced histologist.

The histological presence of damage to the physeal plate or adjacent bone was registered for each slice.

The procedure for the preparation of the sections planned for microscopy has previously been shown not to induce any structural changes (Baranto et al. 2005a; Baranto et al. 2005b), as was further confirmed for the control specimen.

## Results

### Macroscopic features

The macroscopic features of the specimens are shown in Table 1.

**Table 1:** **Macroscopic features of the specimens**

**Specimen**	**A-P diameter (mm)**	**M-L diameter (mm)**	**Footprint distance from epiphyseal line (mm)**	**Macroscopic damage present**
1	35	35	7	No
2	38	39	7	No
3	37	38	7	No
4	38	38	14	No
5	38	38	9	No
6	36	36	5	No
Control	37	38	NA	No
Average	37	37.4		

A-P; anterior-posterior.

M-L; medial-lateral.

Caput diameters ranged from 35 × 35 mm to 38 × 39 mm (average 37 × 37.3 mm). In the specimens loaded vertically, the footprint was 7 mm from the physeal plate. In the specimens loaded in 45° of flexion, the distance of the footprint from the physeal plate varied between 5 and 14 mm.

No macroscopic damage was visible or palpable on any of the specimens after loading.

### Histological findings

**Figure 5:**
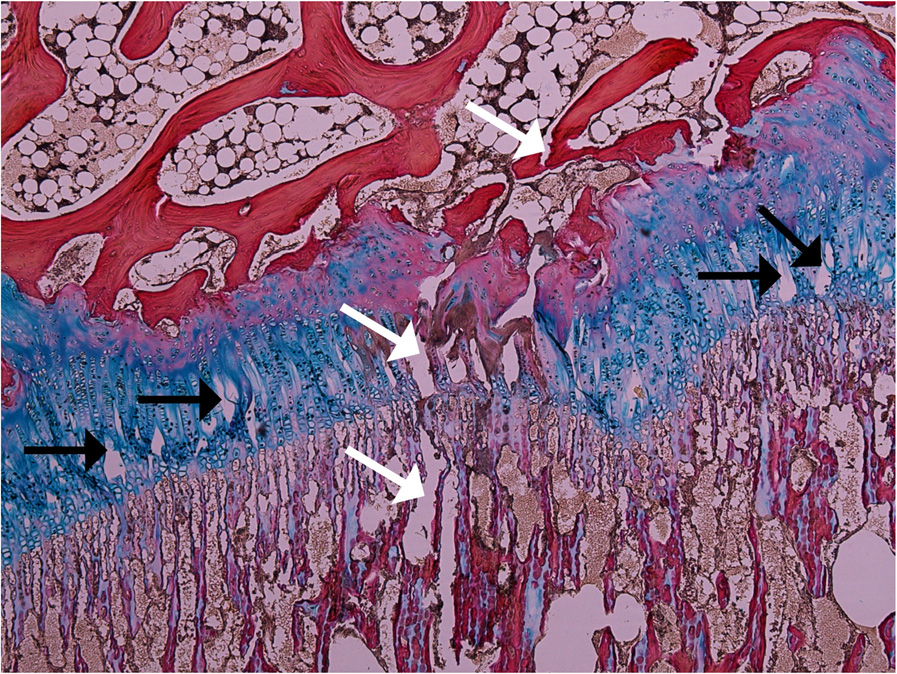
**In the vertically loaded specimens the injuries of the physeal line (black arrows) lay perpendicular to the physeal line, parallel to the cellular columns of the physeal line.** On this microscopic picture, a fracture (white arrows) extending from the epiphyseal bone (above) through the physeal line and into metaphyseal bone (below) is seen.

**Figure 6:**
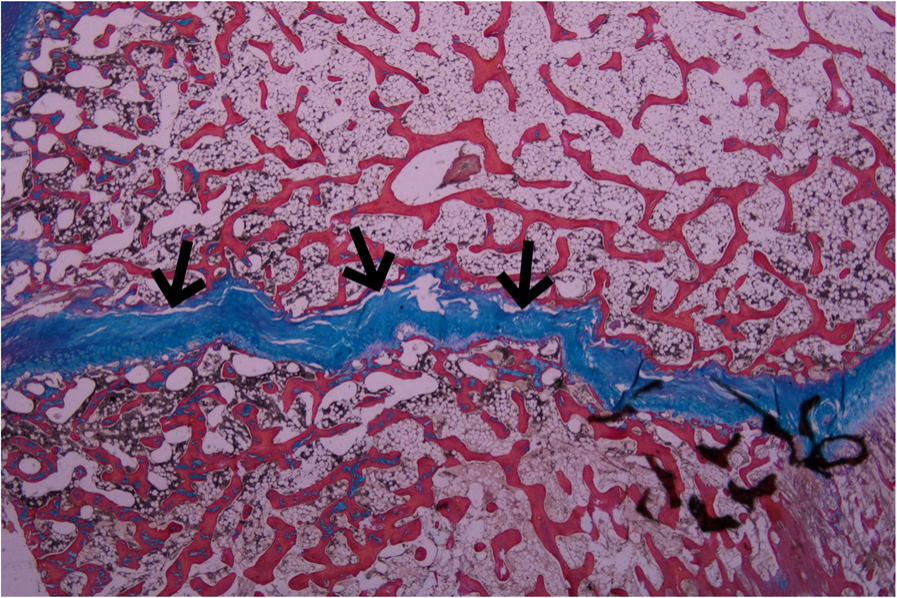
**Microscopic photograph of an antero-superiorly loaded specimen.** In the antero-superiorly loaded specimens the injuries lay parallel to the physeal line (black arrows).

**Figure 7:**
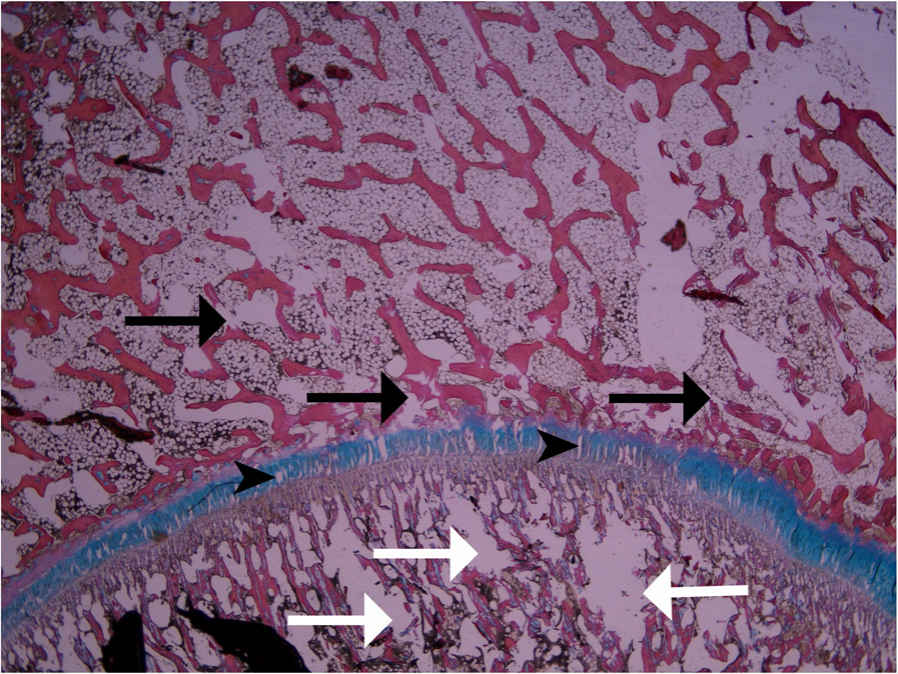
**A microscopic photograph of a vertically loaded specimen.** Fractures of the epiphyseal bone (above, black arrows) and metaphyseal bone (below, white arrows) are seen and the injuries in the physeal line (black arrowheads) are aligned parallel with the cellular columns of the physeal line.

**Figure 8:**
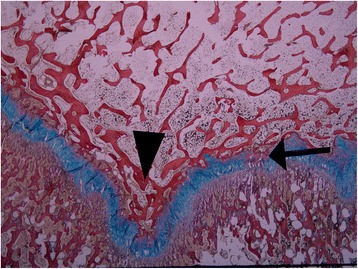
**A microscopic picture of the epiphyseal tubercle (black arrowhead) in a vertically loaded specimen.** The bone density is higher around the tubercle and injuries (black arrow) were often seen in the zone were the bone becomes less dense.

Damage to the physeal plate was seen in all specimens. No injuries were seen in the control. In the vertically loaded specimens, the damage tended to run parallel to the cellular columns of the physeal plate and perpendicularly to the physeal plate (Figure 5). In contrast, in the antero-superiorly loaded specimens, the damage ran parallel to the physeal plate (Figure 6). Microscopic fractures of the adjacent bone were seen in all vertically loaded specimens and in one of those loaded antero-superiorly (Figure 7). Generally, more damage was seen in the slides from the vertically loaded specimens compared with those loaded antero-superiorly. Microscopic fractures, whether in the epiphyseal bone or metaphyseal bone, tended to concentrate laterally in the epiphyseal tubercle, where the bone density was higher than peripherally (Figure 8). The bone density was evaluated based on the light microscopic inspection of stained sections, disclosing a distinct difference in bone structure on either side of the epiphyseal tubercle.

#### Vertically loaded specimens

In specimen 1, three of four slides showed physeal plate injury and microscopic fractures of both the epiphyseal and metaphyseal bone. No damage was seen on the fourth slide.

In specimen 2, two of four slides showed physeal plate injury and microscopic fractures of the metaphyseal bone. On the remaining two slides, no damage was seen.

In specimen 3, two slides showed injuries to the physeal plate and, in one of them, microscopic fractures were also seen in the epiphyseal bone. One slide showed no injuries, while, in one, artefacts made assessment difficult.

#### Antero-superiorly loaded specimens

In specimen 4, three of four slides showed injuries to the physeal plate. No damage was seen on the remaining slide.

In specimen 5, microscopic fractures in the epiphyseal bone and physeal plate injury were seen on one slide. No damage was seen on two slides and, in one, artefacts made assessment difficult.

In specimen 6, physeal plate damage was seen on one slide, while no injuries were seen on the remaining three slides.

### MRI findings

A summary of the MRI findings in the specimens is presented in Table 2.

**Table 2:** **MRI findings of the different zones of femoral head in the specimens**

**Specimen**	**Zones**							
	**1**	**2**	**3**	**4**	**5**	**6**	**7**	**8**
		**Adjacent to the physeal plate**			**Epiphysis**		**Metaphysis**	
	**Superior**		**Inferior**					
	**Medially**	**Laterally**	**Medially**	**Laterally**	**Medially**	**Laterally**	**Medially**	**Laterally**
1	-	-	+	+	0	0	0	0
2	-	+	+	0	0	0	0	0
3	-	0	+	+	0	0	0	0
4	0	0	+	0	0	0	+	+
5	-	0	+	-	0	0	0	0
6	-	0	0	0	0	-	0	0
Control	0	0	0	0	0	0	0	0

+ and – indicate a higher and a lower signal respectively, compared with the control.

**Figure 9:**
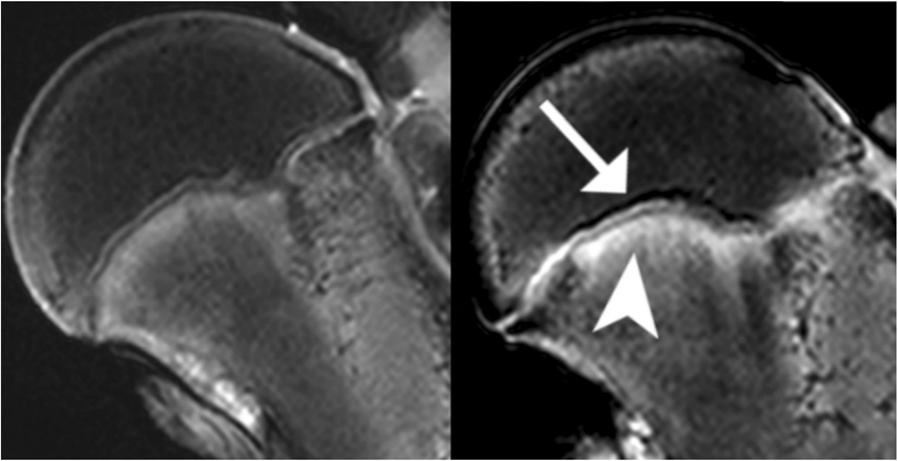
**MRI image of the control hip (left) and a vertically loaded hip (right).** After loading, difference in signal intensity was seen medially to the epiphyseal tubercle. A lower signal was seen above the epiphyseal line (white arrow) and higher signal below the epiphyseal line (white arrowhead).

As compared with the control specimen, differences were seen adjacent to the physeal plate, medially to the tubercle, in the majority of specimens (5 of 6). A reduced signal was demonstrated above the line and an increased signal below the physeal plate (Figure 9). In specimen 4, an increased signal was seen in the metaphysis and a reduced signal was seen laterally in the epiphysis of specimen 6.

No fractures or separations/avulsions were found in any specimen on MRI.

## Discussion

The main finding in the present study was that, after repetitive sub-maximum loading of the young porcine proximal femur, microscopic injuries to the physeal plate and adjacent bone were demonstrable. All specimens showed histological physeal plate injuries and fractures in the adjacent bone were seen in four of six specimens. On MRI, a reduced fluid signal was seen adjacent to the physeal plate in all cases.

The age and size of the samples were comparable to those in a previous study in which the proximal femora were loaded to failure (Jonasson et al. 2014). Hip-joint loading has been shown to reach levels of up to nine times body weight during different daily activities (Bergmann et al. 2001; Bergmann et al. 1993, 2004; Cleather et al. 2013). In this study, we decided to apply about 35-40% of the failure load reported in the static strength study (Jonasson et al. 2014). As no static failure load data were available, the failure load of the antero-superiorly loaded specimens was estimated to be between the vertical failure load (7,273 N) and the anterior failure load (1,728 N). This resulted in 2,500 N (equivalent to 255 kg) for the vertically loaded specimens (35% failure load) and 2,000 N (equivalent to 204 kg) for the antero-superiorly loaded specimens (40% estimated failure load). The specimens used in this study came from animals weighing about 90 kg. The load levels that were chosen are more than twice the body weight (2.2-2.7). Weight distribution in a pig is approximately 60% front and 40% rear limbs (Meijer et al. 2014). This results in a load that is 5.5 to 6.75 times the rear limb weight distribution. However, it is important to recognise that the stance load is not equivalent to the hip-joint load. To our knowledge, no data that quantify the load and movement in the porcine hip joint during daily activity have been presented. Based on estimations of the load to which a human hip joint is subjected during activity, it is believed that the load levels chosen in the present study are reasonable and perhaps even under-estimated. The size and shear strength of the specimens are comparable to those of a 10- to 12-year-old child (Chung et al. 1976; Ipsen et al. 2002). In a previous study in which cyclical loading was applied for 20,000 cycles to the porcine spine, no differences were seen in failure load compared with non-cyclically loaded spines (Thoreson et al. 2010). In the present study, 50,000 cycles were applied; this is approximately comparable to running four marathons for a person of average height or cumulative exposure during one week of top athletic activity. Although comparisons between the porcine model used in the present study and the conditions in the human hip are open to debate, we conclude that the load applied in this study is well within the physiological range possible during sporting activities.

It is not known how loading forces vary in the hip joint during hip movements. Biomechanical studies have shown that, during routine activities, vertical load dominates (Bergmann et al. 2004). What happens in deep flexion of the hip is so far not known. The joint capsule, ligaments and muscles around the hip joint probably prevent changes in loading directions to a certain degree. The extreme range of motion performed during sporting activities and the load applied simultaneously may create unfavourable forces over the physeal plate, creating microscopic injuries that could lead to growth disturbance.

MRI of dead tissue has certain limitations and interpreting the findings is therefore difficult. A difference in fluid distribution was noted within the tissue. Mechanical loading of the tissue probably alters the distribution of fluid. With heavier loads, more redistribution of fluid can occur. Differences in signal intensity between loaded specimens and controls were predominantly observed adjacent to the physeal plate, medially to the epiphyseal tubercle. No separations or injuries could be seen in the cartilage and no fractures were observed, probably due to the poor ability of MRI to detect microfractures. Perhaps micro-CT analysis of the whole specimen could show fracture injuries in the skeletal part of the femur.

Although no macroscopic injuries to the specimens were detected on examination after loading, all specimens showed signs of microscopic injuries. The signs were often subtle and might produce only a few symptoms or even be asymptomatic in an individual (Kaeding and Miller 2013). In the vertically loaded specimens, injuries were seen both in and adjacent to the physeal plate, while physeal plate injuries predominated in the antero-superiorly loaded specimens. The physeal plate has previously been shown to be sensitive to shearing forces (Jonasson et al. 2014). It is possible to speculate that more shearing forces are applied to the physeal plate in the antero-superiorly loaded specimens and this explains the difference in injury patterns between loading directions.

Differences on MRI were predominantly medial to the epiphyseal tubercle, while microscopic injuries were predominantly found laterally to the epiphyseal tubercle. This could be explained by the stabilising function of the epiphyseal tubercle (Jonasson et al. 2014; Tayton 2009), causing the majority of the load to be distributed laterally to the tubercle, thereby causing fractures, while the minority of the load is evenly distributed medially, causing only fluid redistribution.

The cam deformity is located laterally to the epiphyseal tubercle. It is believed to develop during growth in adolescents and appears to be an extension of the physis (Siebenrock et al. 2011; Siebenrock et al. 2004; Agricola et al. 2014). The findings in the present study support the hypothesis that repeated high loads on the adolescent hip can lead to growth disturbance, due to physeal injury and microfractures in the trabecular bone, and consequently the development of cam deformity.

The present study has certain limitations. Relating results from an *in-vitro*, animal model, to the *in-vivo*, human condition is and always will be debatable. Porcine models are commonly used in studies of the hip and spine and in paediatric orthopaedics (Baranto et al. 2005a; Baranto et al. 2005b; Dodds et al. 2008; Hosalkar et al. 2011; Jonasson et al. 2014; Kishan et al. 2006; Pawaskar et al. 2011; Thoreson et al. 2010; Upasani et al. 2006; Wenger et al. 2007; Lundin et al. 2000; Karlsson et al. 1998; Kaigle et al. 1998) and are accepted in the literature as appropriate models (Pearce et al. 2007).

We conclude that the loads applied were within physiological limits. The number of cycles can be regarded as high, but the amount of training performed by many elite athletes is fairly severe, with more than 20 hours’ training every week. Further research is needed to investigate the effect of load level and the number of load cycles on the fatigue properties of the femoral head.

## Conclusion

*In-vitro* cyclical, physiological loading of the young porcine hip leads to histological injuries in and adjacent to the physeal plate. We propose that these injuries are likely to cause growth disturbances seen in adolescent athletes. These injures are also seen on MRI and offer a plausible explanation for the development of the cam deformity of the hip.
